# *fac*-Aqua­(2-carboxy­ethyl-κ^2^ *C*,*O*)trichlorido­tin(IV)–1,4,7,10,13-penta­oxacyclo­penta­deca­ne–water (1/1/2)

**DOI:** 10.1107/S1600536810011633

**Published:** 2010-04-10

**Authors:** Edward R. T. Tiekink, James L. Wardell, Solange M. S. V. Wardell

**Affiliations:** a Department of Chemistry, University of Malaya, 50603 Kuala Lumpur, Malaysia; b Centro de Desenvolvimento Tecnológico em Saúde (CDTS), Fundação Oswaldo Cruz (FIOCRUZ), Casa Amarela, Campus de Manguinhos, Av. Brasil 4365, 21040-900 Rio de Janeiro, RJ, Brazil; c CHEMSOL, 1 Harcourt Road, Aberdeen AB15 5NY, Scotland

## Abstract

In the title compound, [Sn(C_3_H_5_O_2_)Cl_3_(H_2_O)]·C_10_H_20_O_5_·2H_2_O, the Sn^IV^ atom is octa­hedrally coordinated within a *fac*-CO_2_Cl_3_ donor set, arising from the *C*,*O*-bidentate carboxy­ethyl ligand, a water mol­ecule and three chloride ligands. In the crystal, supra­molecular chains linked by O—H⋯O hydrogen bonds propagate along the *c* axis These chains are connected into layers in the *ac* plane *via* C—H⋯O inter­actions.

## Related literature

For original industrial inter­est in functionally substituted alk­yl–tin compounds, see: Lanigen & Weinberg (1976 ▶). For studies concerning the coordination chemistry of functionally substituted alk­yl–tin compounds, see: Harrison *et al.* (1979 ▶); Howie *et al.* (1986 ▶); Balasubramanian *et al.* (1997 ▶); Tian *et al.* (2005 ▶); de Lima *et al.* (2009 ▶). For related structures of functionally substituted alk­yl–tin compounds, see: Buchanan *et al.* (1996 ▶); Howie & Wardell (2001 ▶, 2002 ▶). For a review on tin–crown ether compounds, see: Cusack & Smith (1990 ▶). For related structures of organotin(IV) and tin(IV) halide complexes with crown ethers, see: Barnes & Weakley (1976 ▶); Cusack *et al.* (1984 ▶); Amini *et al.* (1984 ▶, 2002 ▶); Russo *et al.* (1984 ▶); Valle *et al.* (1984 ▶, 1985 ▶); Rivarola *et al.* (1986 ▶); Hough *et al.* (1986 ▶); Bott *et al.* (1987 ▶); Mitra *et al.* (1993 ▶); Yap *et al.* (1996 ▶); Wolff *et al.* (2009 ▶); Wardell *et al.* (2010 ▶). For a related tin compound with a 2-carboxy­ethyl ligand, see: Somphon *et al.* (2006 ▶). For the synthesis of MeO_2_CCH_2_CH_2_CO_2_SnCl_3_, see: Hutton & Oakes (1976 ▶).
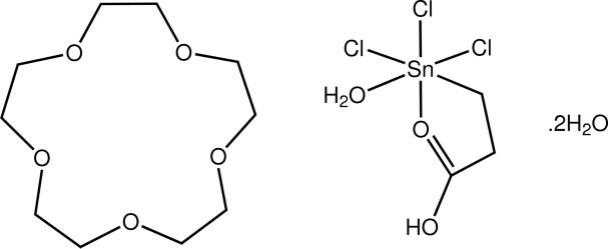


## Experimental

### 

#### Crystal data

[Sn(C_3_H_5_O_2_)Cl_3_(H_2_O)]·C_10_H_20_O_5_·2H_2_O
*M* *_r_* = 572.42
Monoclinic, 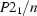

*a* = 7.2193 (2) Å
*b* = 29.6516 (13) Å
*c* = 10.3871 (5) Å
β = 91.857 (2)°
*V* = 2222.33 (16) Å^3^
*Z* = 4
Mo *K*α radiation
μ = 1.56 mm^−1^
*T* = 120 K
0.42 × 0.20 × 0.07 mm

#### Data collection

Nonius KappaCCD diffractometer
Absorption correction: multi-scan (*SADABS*; Sheldrick, 2007 ▶) *T*_min_ = 0.621, *T*_max_ = 0.746
12758 measured reflections
3721 independent reflections
3241 reflections with *I* > 2σ(*I*)
*R*_int_ = 0.037

#### Refinement

*R*[*F* ^2^ > 2σ(*F* ^2^)] = 0.027
*wR*(*F* ^2^) = 0.088
*S* = 1.19
3721 reflections
265 parameters
10 restraints
H atoms treated by a mixture of independent and constrained refinement
Δρ_max_ = 0.70 e Å^−3^
Δρ_min_ = −0.74 e Å^−3^

### 

Data collection: *COLLECT* (Hooft, 1998 ▶); cell refinement: *DENZO* (Otwinowski & Minor, 1997 ▶) and *COLLECT*; data reduction: *DENZO* and *COLLECT*; program(s) used to solve structure: *SHELXS97* (Sheldrick, 2008 ▶); program(s) used to refine structure: *SHELXL97* (Sheldrick, 2008 ▶); molecular graphics: *ORTEP-3* (Farrugia, 1997 ▶), *DIAMOND* (Brandenburg, 2006 ▶); software used to prepare material for publication: *publCIF* (Westrip, 2010 ▶).

## Supplementary Material

Crystal structure: contains datablocks global, I. DOI: 10.1107/S1600536810011633/hb5380sup1.cif

Structure factors: contains datablocks I. DOI: 10.1107/S1600536810011633/hb5380Isup2.hkl

Additional supplementary materials:  crystallographic information; 3D view; checkCIF report

## Figures and Tables

**Table 1:** Selected bond lengths (Å)

Sn—C1	2.148 (3)
Sn—O1	2.284 (2)
Sn—O1w	2.234 (2)
Sn—Cl1	2.4287 (9)
Sn—Cl2	2.4014 (9)
Sn—Cl3	2.3706 (8)

**Table 2:** Hydrogen-bond geometry (Å, °)

*D*—H⋯*A*	*D*—H	H⋯*A*	*D*⋯*A*	*D*—H⋯*A*
O2—H1o⋯O2w	0.84 (3)	1.71 (3)	2.551 (3)	172 (4)
O1w—H1w⋯O3w	0.84 (2)	1.85 (3)	2.640 (3)	156 (3)
O1w—H2w⋯O4	0.84 (3)	1.88 (2)	2.686 (3)	161 (3)
O2w—H3w⋯O3^i^	0.84 (2)	1.89 (1)	2.720 (3)	172 (3)
O2w—H4w⋯O6^i^	0.84 (2)	1.92 (2)	2.752 (3)	169 (3)
O3w—H5w⋯O7	0.84 (2)	2.02 (3)	2.827 (3)	162 (3)
O3w—H6w⋯O5	0.84 (3)	1.91 (3)	2.744 (3)	172 (3)
C8—H8b⋯O2^ii^	0.99	2.52	3.491 (4)	165
C12—H12b⋯O3w^iii^	0.99	2.42	3.266 (5)	143

Symmetry codes: (i) 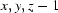; (ii) 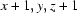; (iii) 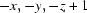.

**Fig. 1.**
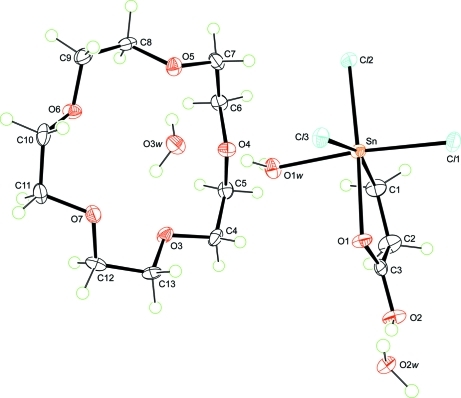
The molecular structures of (I) showing displacement ellipsoids at the 50% probability level.

**Fig. 2.**
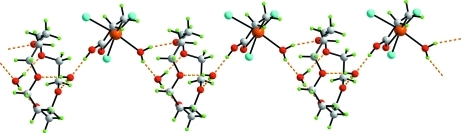
A view of the supramolecular chain aligned along the c axis in the crystal structure of (I) formed through the agency of O–H···O hydrogen bonding interactions (orange dashed lines).

**Fig. 3.**
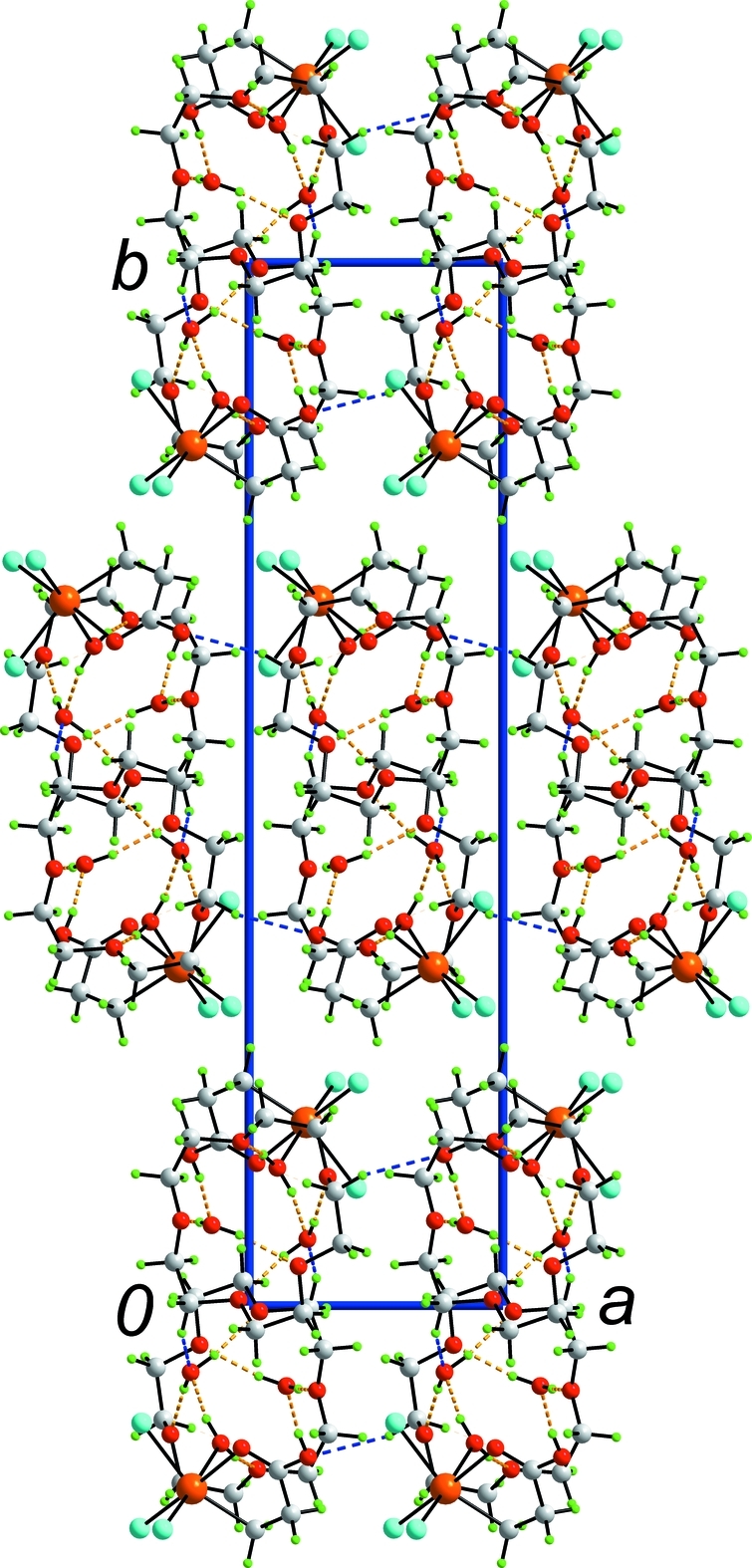
A view of the unit cell contents in (I) shown in projection down the c axis and highlighting the C–H···O interactions (blue dashed lines) formed between the chains to form two-dimensional arrays that stack along the b axis; O–H···O hydrogen bonds are shown as orange dashed lines.

## supplementary crystallographic information

### Comment

The so-called, estertin chlorides, RO_2_CCH_2_CH_2_SnCl_3_, as well as the diestertin dichlorides (RO_2_CCH_2_CH_2_)_2_SnCl_2_ (R = Me, Et, *etc*.), were initially made in the 1970's (Hutton & Oakes, 1976) as precursors of organotin mercaptide PVC stabilizers by AZKO Chemie (Lanigen & Weinberg, 1976). This intention has never (yet) been fulfilled industrially. However, interest in the coordination chemistry of such compounds has been maintained until today, with particular interest centering on the coordinating mode of the RO_2_CCH_2_CH_2_ ligand, *i.e*. whether mono-or bi-dentate (Tian *et al.*, 2005; Balasubramanian *et al.*, 1997; Harrison *et al.*, 1979; de Lima *et al.*, 2009; Buchanan *et al.*, 1996; Howie & Wardell, 2001; Howie & Wardell, 2002; Howie *et al.*, 1986). We now wish to report the structure of *fac*-aqua(2-carboxyethyl-κ^2^*C*,*O*)trichloridotin(IV) 1,4,7,10,13-pentaoxacyclopentadecane dihydrate, (I). Crown ether complexes of tin and organotin halides have been variously reported (Barnes & Weakley, 1976; Cusack *et al.*, 1984; Amini *et al.* 1984; Amini *et al.* 2002; Russo *et al.*, 1984; Valle *et al.*, 1984, 1985; Rivarola *et al.* 1986; Hough *et al.*, 1986; Bott *et al.*, 1987; Cusack & Smith, 1990; Mitra *et al.*, 1993); Yap *et al.*, 1996; Wolff *et al.*, 2009; Wardell *et al.*, 2010).

The asymmetric unit of (I) comprises an organotin molecule, a 15-crown-5 molecule and two solvent water molecules of crystallisation, Fig. 1. The tin atom exists within a *fac*-CCl_3_O_2_ donor set defined by three Cl atoms, chelating C- and O- atoms from the 2-carboxyethyl ligand, and a coordinated water molecule. The C3–O1 [1.233 (4) Å] and C3–O2 [1.289 (4) Å] bond distances, and the pattern on intermolecular hydrogen bonds (see below) indicate the coordination of the carbonyl-O1 atom. The four non-hydrogen atoms of the chelating ligand are planar with the C1–C2–C3–O1 torsion angle being 0.5 (5) °. However, the five-membered chelate ring is not planar as the tin atom lies above the plane through the chelating ligand as indicated in the values of the Sn–C1–C2–C3 and Sn–O1–C3–C2 torsion angles of 9.1 (4) and -9.3 (4) °, respectively. There is only one other tin structure containing a 2-carboxyethyl ligand available in the literature and this adopts the same mode of coordination (Somphon *et al.*, 2006). The Sn–Cl bond distances span a large range, Table 1, with the shorter Sn—Cl3 bond having the Cl3 atom *trans* to the C atom of the organic ligand. The longer Sn—Cl1 bond has the Cl1 atom *trans* to the aqua ligand which forms a shorter Sn–O1w bond distance than the dative Sn–O1 bond, Table 1.

There are a large number of O–H···O hydrogen bonding interactions in the crystal structure of (I), Table 2. One of the H atoms of the aqua ligand forms a hydrogen bond with a lattice water (O3w) molecule and the other H atom is connected to an ether-O atom. Each of the H atoms of the O3w water molecule is connected to an ether-O atom. As a result, a nine-membered {···HOH···OH···OC_2_O} synthon is formed, Fig. 2. The hydroxyl group forms a hydrogen bond to the second lattice water molecule which, like the O3w water molecule, forms two donor interactions to ether-O atoms so that each ether-O atom participates in the hydrogen bonding scheme. The hydrogen bonds lead to the formation of supramolecular chains along the *c* axis, Fig. 2. Chains are linked into layers in the *ac* plane via C–H···O interactions, Table 2 and Fig. 3.

### Experimental

The title compound was obtained from a solution of MeO_2_CCH_2_CH_2_CO_2_SnCl_3_ (0.360 g, 1 mmol), obtained from SnCl_2_, H_2_C=CHCO_2_Me and HCl (Hutton & Oakes, 1976), and 15-crown-5 (0.220 g, 1 mmol) in MeOH (20 ml). The solution was gently heated for 30 minutes and maintained at room temperature and colourless blades of (I) were harvested after 4 days. m.pt. 423-426 K. IR: ν 1654 (C=O) cm^-1^.

### Refinement

The C-bound H atoms were geometrically placed (C–H = 0.99 Å) and refined as riding with *U_iso_*(H) = 1.2*U_eq_*(parent atom). The O—H atoms were refined with the distance restraint 0.840±0.001 Å, and with *U_iso_*(H) = 1.5*U_eq_*(parent atom).

### Crystal data

**Table:**

[Sn(C_3_H_5_O_2_)Cl_3_(H_2_O)]·C_10_H_20_O_5_·2H_2_O	*F*(000) = 1160
*M**_r_* = 572.42	*D*_x_ = 1.711 Mg m^−^^3^
Monoclinic, *P*2_1_/*n*	Mo *K*α radiation, λ = 0.71073 Å
Hall symbol: -P 2yn	Cell parameters from 15234 reflections
*a* = 7.2193 (2) Å	θ = 2.9–27.5°
*b* = 29.6516 (13) Å	µ = 1.56 mm^−^^1^
*c* = 10.3871 (5) Å	*T* = 120 K
β = 91.857 (2)°	Blade, colourless
*V* = 2222.33 (16) Å^3^	0.42 × 0.20 × 0.07 mm
*Z* = 4	

### Data collection

**Table:**

Nonius KappaCCD diffractometer	3721 independent reflections
Radiation source: Enraf Nonius FR591 rotating anode	3241 reflections with *I* > 2σ(*I*)
10 cm confocal mirrors	*R*_int_ = 0.037
Detector resolution: 9.091 pixels mm^-1^	θ_max_ = 25.0°, θ_min_ = 3.1°
φ and ω scans	*h* = −8→8
Absorption correction: multi-scan (*SADABS*; Sheldrick, 2007)	*k* = −35→35
*T*_min_ = 0.621, *T*_max_ = 0.746	*l* = −12→12
12758 measured reflections	

### Refinement

**Table:**

Refinement on *F*^2^	Primary atom site location: structure-invariant direct methods
Least-squares matrix: full	Secondary atom site location: difference Fourier map
*R*[*F*^2^ > 2σ(*F*^2^)] = 0.027	Hydrogen site location: inferred from neighbouring sites
*wR*(*F*^2^) = 0.088	H atoms treated by a mixture of independent and constrained refinement
*S* = 1.19	*w* = 1/[σ^2^(*F*_o_^2^) + (0.0465*P*)^2^ + 0.1546*P*] where *P* = (*F*_o_^2^ + 2*F*_c_^2^)/3
3721 reflections	(Δ/σ)_max_ = 0.001
265 parameters	Δρ_max_ = 0.70 e Å^−^^3^
10 restraints	Δρ_min_ = −0.74 e Å^−^^3^

### Special details

**Table:**

Geometry. All s.u.'s (except the s.u. in the dihedral angle between two l.s. planes) are estimated using the full covariance matrix. The cell s.u.'s are taken into account individually in the estimation of s.u.'s in distances, angles and torsion angles; correlations between s.u.'s in cell parameters are only used when they are defined by crystal symmetry. An approximate (isotropic) treatment of cell s.u.'s is used for estimating s.u.'s involving l.s. planes.
Refinement. Refinement of *F*^2^ against ALL reflections. The weighted *R*-factor wR and goodness of fit *S* are based on *F*^2^, conventional *R*-factors *R* are based on *F*, with *F* set to zero for negative *F*^2^. The threshold expression of *F*^2^ > 2σ(*F*^2^) is used only for calculating *R*-factors(gt) etc. and is not relevant to the choice of reflections for refinement. *R*-factors based on *F*^2^ are statistically about twice as large as those based on *F*, and *R*- factors based on ALL data will be even larger.

### Fractional atomic coordinates and isotropic or equivalent isotropic displacement parameters (Å^2^)

**Table:**

	*x*	*y*	*z*	*U*_iso_*/*U*_eq_	
Sn	0.22564 (3)	0.175614 (7)	0.29201 (2)	0.01417 (11)	
Cl1	0.34019 (12)	0.21445 (3)	0.10473 (9)	0.0209 (2)	
Cl2	0.43021 (12)	0.21340 (3)	0.44366 (9)	0.0211 (2)	
Cl3	0.42241 (11)	0.11255 (3)	0.26495 (9)	0.0186 (2)	
O1	0.0296 (3)	0.13662 (7)	0.1555 (2)	0.0178 (5)	
O2	−0.2328 (3)	0.14512 (9)	0.0433 (2)	0.0225 (6)	
H1O	−0.195 (5)	0.1229 (8)	0.001 (3)	0.034*	
O3	−0.2678 (3)	0.08134 (8)	0.6564 (2)	0.0199 (6)	
O4	−0.0320 (3)	0.15697 (8)	0.6706 (2)	0.0187 (6)	
O5	0.3096 (3)	0.12348 (8)	0.7855 (2)	0.0196 (6)	
O6	0.1948 (3)	0.03774 (8)	0.8827 (2)	0.0198 (6)	
O7	−0.0411 (3)	0.00568 (8)	0.6891 (2)	0.0194 (6)	
O1W	0.1114 (3)	0.13208 (8)	0.4460 (2)	0.0204 (6)	
H1W	0.168 (4)	0.1089 (7)	0.473 (3)	0.031*	
H2W	0.056 (4)	0.1448 (10)	0.506 (2)	0.031*	
O2W	−0.1475 (3)	0.07709 (8)	−0.0930 (2)	0.0183 (5)	
H3W	−0.194 (4)	0.0799 (13)	−0.1676 (11)	0.027*	
H4W	−0.0378 (18)	0.0677 (12)	−0.094 (3)	0.027*	
O3W	0.2298 (3)	0.06380 (9)	0.5896 (2)	0.0228 (6)	
H5W	0.137 (3)	0.0475 (10)	0.603 (3)	0.034*	
H6W	0.256 (4)	0.0798 (11)	0.654 (2)	0.034*	
C1	−0.0237 (5)	0.21467 (12)	0.3046 (4)	0.0225 (9)	
H1A	−0.0752	0.2109	0.3911	0.027*	
H1B	0.0051	0.2470	0.2924	0.027*	
C2	−0.1649 (5)	0.19982 (13)	0.2036 (4)	0.0255 (9)	
H2A	−0.1831	0.2245	0.1404	0.031*	
H2B	−0.2845	0.1949	0.2454	0.031*	
C3	−0.1152 (5)	0.15757 (12)	0.1323 (3)	0.0163 (8)	
C4	−0.3106 (5)	0.12264 (12)	0.5911 (4)	0.0195 (8)	
H4A	−0.4466	0.1262	0.5800	0.023*	
H4B	−0.2561	0.1227	0.5048	0.023*	
C5	−0.2309 (4)	0.16057 (12)	0.6714 (4)	0.0201 (8)	
H5A	−0.2713	0.1900	0.6351	0.024*	
H5B	−0.2740	0.1584	0.7607	0.024*	
C6	0.0615 (5)	0.18000 (11)	0.7739 (4)	0.0198 (8)	
H6A	0.0135	0.1697	0.8571	0.024*	
H6B	0.0398	0.2129	0.7661	0.024*	
C7	0.2661 (5)	0.17016 (12)	0.7691 (4)	0.0205 (8)	
H7A	0.3118	0.1804	0.6851	0.025*	
H7B	0.3322	0.1877	0.8374	0.025*	
C8	0.3375 (5)	0.10950 (12)	0.9175 (4)	0.0197 (8)	
H8A	0.2278	0.1173	0.9678	0.024*	
H8B	0.4471	0.1249	0.9568	0.024*	
C9	0.3668 (4)	0.05926 (12)	0.9177 (4)	0.0216 (8)	
H9A	0.4623	0.0511	0.8554	0.026*	
H9B	0.4101	0.0491	1.0044	0.026*	
C10	0.2127 (5)	−0.00743 (12)	0.8347 (4)	0.0236 (9)	
H10A	0.2632	−0.0275	0.9034	0.028*	
H10B	0.2982	−0.0079	0.7620	0.028*	
C11	0.0241 (5)	−0.02314 (12)	0.7904 (4)	0.0219 (8)	
H11A	0.0305	−0.0546	0.7593	0.026*	
H11B	−0.0617	−0.0221	0.8628	0.026*	
C12	−0.2365 (4)	0.00224 (12)	0.6619 (4)	0.0201 (8)	
H12A	−0.3045	0.0023	0.7431	0.024*	
H12B	−0.2649	−0.0262	0.6153	0.024*	
C13	−0.2938 (5)	0.04203 (12)	0.5804 (4)	0.0192 (8)	
H13A	−0.2174	0.0437	0.5031	0.023*	
H13B	−0.4255	0.0392	0.5520	0.023*	

### Atomic displacement parameters (Å^2^)

**Table:**

	*U*^11^	*U*^22^	*U*^33^	*U*^12^	*U*^13^	*U*^23^
Sn	0.01453 (16)	0.01367 (15)	0.01420 (17)	0.00086 (9)	−0.00119 (11)	−0.00125 (10)
Cl1	0.0302 (5)	0.0175 (4)	0.0149 (5)	−0.0001 (4)	−0.0009 (4)	0.0030 (4)
Cl2	0.0254 (5)	0.0216 (5)	0.0161 (5)	−0.0061 (4)	−0.0053 (4)	0.0014 (4)
Cl3	0.0191 (4)	0.0172 (4)	0.0195 (5)	0.0051 (3)	0.0019 (4)	0.0019 (4)
O1	0.0169 (13)	0.0175 (12)	0.0188 (14)	0.0026 (10)	−0.0062 (10)	−0.0055 (11)
O2	0.0168 (13)	0.0307 (15)	0.0196 (15)	0.0026 (11)	−0.0035 (11)	−0.0100 (13)
O3	0.0264 (14)	0.0175 (13)	0.0155 (14)	0.0019 (10)	−0.0053 (11)	−0.0021 (11)
O4	0.0169 (13)	0.0208 (13)	0.0184 (15)	−0.0001 (10)	0.0010 (11)	−0.0043 (12)
O5	0.0248 (13)	0.0202 (13)	0.0138 (15)	−0.0001 (10)	0.0028 (11)	−0.0006 (11)
O6	0.0137 (12)	0.0207 (13)	0.0248 (15)	0.0007 (10)	−0.0010 (11)	−0.0024 (11)
O7	0.0178 (13)	0.0166 (12)	0.0240 (15)	−0.0002 (10)	0.0019 (11)	0.0037 (11)
O1W	0.0256 (14)	0.0191 (13)	0.0169 (14)	0.0009 (11)	0.0074 (11)	−0.0007 (12)
O2W	0.0178 (13)	0.0218 (13)	0.0149 (14)	0.0031 (11)	−0.0034 (10)	−0.0008 (12)
O3W	0.0274 (15)	0.0230 (14)	0.0183 (15)	−0.0063 (11)	0.0049 (12)	−0.0037 (12)
C1	0.0188 (19)	0.023 (2)	0.026 (2)	0.0051 (15)	−0.0010 (16)	−0.0038 (17)
C2	0.0254 (19)	0.026 (2)	0.025 (2)	0.0078 (17)	−0.0026 (17)	−0.0091 (18)
C3	0.0143 (18)	0.0206 (19)	0.014 (2)	−0.0030 (15)	0.0047 (15)	0.0044 (17)
C4	0.0179 (18)	0.024 (2)	0.017 (2)	0.0047 (15)	−0.0023 (15)	0.0053 (17)
C5	0.0195 (19)	0.0186 (18)	0.022 (2)	0.0033 (14)	0.0034 (16)	0.0022 (17)
C6	0.028 (2)	0.0159 (18)	0.015 (2)	−0.0033 (15)	0.0020 (16)	−0.0063 (15)
C7	0.025 (2)	0.0186 (19)	0.018 (2)	−0.0076 (15)	0.0036 (16)	0.0002 (16)
C8	0.0129 (18)	0.029 (2)	0.017 (2)	−0.0008 (15)	−0.0035 (15)	−0.0019 (17)
C9	0.0153 (18)	0.029 (2)	0.020 (2)	0.0014 (16)	−0.0012 (15)	0.0031 (18)
C10	0.027 (2)	0.0179 (19)	0.026 (2)	0.0065 (15)	0.0012 (17)	0.0086 (18)
C11	0.028 (2)	0.0155 (18)	0.023 (2)	−0.0010 (15)	0.0019 (16)	0.0061 (17)
C12	0.0172 (19)	0.0192 (19)	0.024 (2)	−0.0057 (14)	0.0019 (16)	−0.0086 (17)
C13	0.0199 (18)	0.0215 (19)	0.016 (2)	−0.0014 (15)	−0.0048 (15)	−0.0078 (16)

### Geometric parameters (Å, °)

**Table:**

Sn—C1	2.148 (3)	C2—C3	1.505 (5)
Sn—O1	2.284 (2)	C2—H2A	0.9900
Sn—O1w	2.234 (2)	C2—H2B	0.9900
Sn—Cl1	2.4287 (9)	C4—C5	1.504 (5)
Sn—Cl2	2.4014 (9)	C4—H4A	0.9900
Sn—Cl3	2.3706 (8)	C4—H4B	0.9900
O1—C3	1.233 (4)	C5—H5A	0.9900
O2—C3	1.289 (4)	C5—H5B	0.9900
O2—H1O	0.84 (3)	C6—C7	1.508 (5)
O3—C13	1.417 (4)	C6—H6A	0.9900
O3—C4	1.429 (4)	C6—H6B	0.9900
O4—C6	1.423 (4)	C7—H7A	0.9900
O4—C5	1.440 (4)	C7—H7B	0.9900
O5—C7	1.428 (4)	C8—C9	1.505 (5)
O5—C8	1.441 (4)	C8—H8A	0.9900
O6—C9	1.433 (4)	C8—H8B	0.9900
O6—C10	1.436 (4)	C9—H9A	0.9900
O7—C11	1.424 (4)	C9—H9B	0.9900
O7—C12	1.433 (4)	C10—C11	1.497 (5)
O1W—H1W	0.84 (2)	C10—H10A	0.9900
O1W—H2W	0.84 (3)	C10—H10B	0.9900
O2W—H3W	0.839 (16)	C11—H11A	0.9900
O2W—H4W	0.840 (17)	C11—H11B	0.9900
O3W—H5W	0.84 (2)	C12—C13	1.502 (5)
O3W—H6W	0.84 (3)	C12—H12A	0.9900
C1—C2	1.504 (5)	C12—H12B	0.9900
C1—H1A	0.9900	C13—H13A	0.9900
C1—H1B	0.9900	C13—H13B	0.9900
			
C1—Sn—O1W	86.48 (12)	O4—C5—H5B	110.2
C1—Sn—O1	78.88 (11)	C4—C5—H5B	110.2
O1W—Sn—O1	85.19 (9)	H5A—C5—H5B	108.5
C1—Sn—Cl3	159.90 (10)	O4—C6—C7	108.9 (3)
O1W—Sn—Cl3	82.27 (6)	O4—C6—H6A	109.9
O1—Sn—Cl3	83.61 (6)	C7—C6—H6A	109.9
C1—Sn—Cl2	101.97 (10)	O4—C6—H6B	109.9
O1W—Sn—Cl2	91.92 (7)	C7—C6—H6B	109.9
O1—Sn—Cl2	176.94 (6)	H6A—C6—H6B	108.3
Cl3—Sn—Cl2	95.03 (3)	O5—C7—C6	113.3 (3)
C1—Sn—Cl1	95.78 (11)	O5—C7—H7A	108.9
O1W—Sn—Cl1	172.17 (7)	C6—C7—H7A	108.9
O1—Sn—Cl1	87.89 (6)	O5—C7—H7B	108.9
Cl3—Sn—Cl1	93.33 (3)	C6—C7—H7B	108.9
Cl2—Sn—Cl1	94.94 (3)	H7A—C7—H7B	107.7
C3—O1—Sn	111.8 (2)	O5—C8—C9	107.5 (3)
C3—O2—H1O	112 (3)	O5—C8—H8A	110.2
C13—O3—C4	114.7 (3)	C9—C8—H8A	110.2
C6—O4—C5	114.1 (3)	O5—C8—H8B	110.2
C7—O5—C8	114.6 (3)	C9—C8—H8B	110.2
C9—O6—C10	114.6 (2)	H8A—C8—H8B	108.5
C11—O7—C12	113.7 (3)	O6—C9—C8	108.7 (3)
Sn—O1W—H1W	121 (2)	O6—C9—H9A	110.0
Sn—O1W—H2W	118 (2)	C8—C9—H9A	110.0
H1W—O1W—H2W	111 (3)	O6—C9—H9B	110.0
H3W—O2W—H4W	112 (3)	C8—C9—H9B	110.0
H5W—O3W—H6W	111 (3)	H9A—C9—H9B	108.3
C2—C1—Sn	110.5 (2)	O6—C10—C11	107.8 (3)
C2—C1—H1A	109.6	O6—C10—H10A	110.1
Sn—C1—H1A	109.6	C11—C10—H10A	110.1
C2—C1—H1B	109.6	O6—C10—H10B	110.1
Sn—C1—H1B	109.6	C11—C10—H10B	110.1
H1A—C1—H1B	108.1	H10A—C10—H10B	108.5
C1—C2—C3	114.8 (3)	O7—C11—C10	108.4 (3)
C1—C2—H2A	108.6	O7—C11—H11A	110.0
C3—C2—H2A	108.6	C10—C11—H11A	110.0
C1—C2—H2B	108.6	O7—C11—H11B	110.0
C3—C2—H2B	108.6	C10—C11—H11B	110.0
H2A—C2—H2B	107.5	H11A—C11—H11B	108.4
O1—C3—O2	122.1 (3)	O7—C12—C13	107.9 (3)
O1—C3—C2	122.5 (3)	O7—C12—H12A	110.1
O2—C3—C2	115.4 (3)	C13—C12—H12A	110.1
O3—C4—C5	107.7 (3)	O7—C12—H12B	110.1
O3—C4—H4A	110.2	C13—C12—H12B	110.1
C5—C4—H4A	110.2	H12A—C12—H12B	108.4
O3—C4—H4B	110.2	O3—C13—C12	107.7 (3)
C5—C4—H4B	110.2	O3—C13—H13A	110.2
H4A—C4—H4B	108.5	C12—C13—H13A	110.2
O4—C5—C4	107.8 (3)	O3—C13—H13B	110.2
O4—C5—H5A	110.2	C12—C13—H13B	110.2
C4—C5—H5A	110.2	H13A—C13—H13B	108.5
			
C1—Sn—O1—C3	10.9 (2)	C13—O3—C4—C5	−165.7 (3)
O1W—Sn—O1—C3	98.2 (2)	C6—O4—C5—C4	−159.8 (3)
Cl3—Sn—O1—C3	−179.1 (2)	O3—C4—C5—O4	67.1 (3)
Cl2—Sn—O1—C3	117.3 (11)	C5—O4—C6—C7	174.9 (3)
Cl1—Sn—O1—C3	−85.5 (2)	C8—O5—C7—C6	−87.7 (4)
O1W—Sn—C1—C2	−95.8 (3)	O4—C6—C7—O5	−62.2 (4)
O1—Sn—C1—C2	−10.0 (3)	C7—O5—C8—C9	175.7 (3)
Cl3—Sn—C1—C2	−39.8 (5)	C10—O6—C9—C8	158.9 (3)
Cl2—Sn—C1—C2	173.0 (2)	O5—C8—C9—O6	−70.4 (3)
Cl1—Sn—C1—C2	76.7 (3)	C9—O6—C10—C11	−174.4 (3)
Sn—C1—C2—C3	9.1 (4)	C12—O7—C11—C10	−164.0 (3)
Sn—O1—C3—O2	169.4 (3)	O6—C10—C11—O7	61.5 (4)
Sn—O1—C3—C2	−9.3 (4)	C11—O7—C12—C13	165.3 (3)
C1—C2—C3—O1	0.5 (5)	C4—O3—C13—C12	178.0 (3)
C1—C2—C3—O2	−178.3 (3)	O7—C12—C13—O3	−65.7 (3)

### Hydrogen-bond geometry (Å, °)

**Table:**

*D*—H···*A*	*D*—H	H···*A*	*D*···*A*	*D*—H···*A*
O2—H1o···O2w	0.84 (3)	1.71 (3)	2.551 (3)	172 (4)
O1w—H1w···O3w	0.84 (2)	1.85 (3)	2.640 (3)	156 (3)
O1w—H2w···O4	0.84 (3)	1.88 (2)	2.686 (3)	161 (3)
O2w—H3w···O3^i^	0.839 (16)	1.888 (14)	2.720 (3)	172 (3)
O2w—H4w···O6^i^	0.840 (17)	1.92 (2)	2.752 (3)	169 (3)
O3w—H5w···O7	0.84 (2)	2.02 (3)	2.827 (3)	162 (3)
O3w—H6w···O5	0.84 (3)	1.91 (3)	2.744 (3)	172 (3)
C8—H8b···O2^ii^	0.99	2.52	3.491 (4)	165
C12—H12b···O3w^iii^	0.99	2.42	3.266 (5)	143

Symmetry codes:  (i) *x*, *y*, *z*−1; (ii) *x*+1, *y*, *z*+1; (iii) −*x*, −*y*, −*z*+1.
